# Surveillance for Enteric Fever in India (SEFI) Collaborators

**DOI:** 10.1093/infdis/jiab527

**Published:** 2021-11-23

## TIER 1 COHORT SURVEILLANCE

KEM Hospital Research Centre, Pune, Maharashtra
  Ashish Bavdekar, MBBS, DCH, DNB
  Ankita Shrivastava, BAMS, MPH
  Sonali Sanghavi, MD, PhD
  Sanjay Juvekar, PhD
  Bharat Choudhari, MA, BEd
  Tathagata Bhattacharjee, BSc, MBA
  Sandeep Bhujbal, BSc, MCM
Indian Council of Medical Research-National Institute of Cholera and Enteric Diseases, Kolkata
  Shanta Dutta, MD, PhD
  Suman Kanungo, MBBS, PhD
  Jayanta Saha, MBBS
  Pranab Chatterjee, MD
Centre for Health Research and Development-Society for Applied Studies, New Delhi
  Temsunaro Rongsen-Chandola, MBBS, PhD
  Bireshwar Sinha, MD
  Nidhi Goyal, MBBS, MSc
  Deepak More, MD
  Alok Arya, MPharm
  Chandra Mohan Kumar, MD
  Ananya Tupaki-Sreepurna, MD
  Ankita Dutta, MSc
  Aparna Chakravarty, MD
  Mohammed Aslam, MBBS
Christian Medical College, Vellore, Tamil Nadu
  Jacob John, MD, PhD
  Manikandan Srinivasan, MD
  Kulandaipalayam Natarajan Sindhu, MD
  Prabakhar D. Moses, MD, FRCP
  Winsley Rose, MD
  Malathi Murugesan, MD
  Sathyapriya Subramaniam, MSc
  Swathi Vajja, MBBS
  Anita Shirley David, MD
  Ranjith Kumar R., MPhil
  Atrayee Nag, MBBS

## TIER 2 HYBRID SURVEILLANCE

Postgraduate Institute of Medical Education and Research, Chandigarh
  Madhu Gupta, MD, PhD
  Adarsh Bansal, MPH
  Bhavneet Bharti, MD
  Vikas Suri, MD
  Vivek Sagar, PhD
  Nayantara, MBBS
  Nitasha Kallyan, MBBS
  Rajesh Kumar, MD
  Krishna Chaudhary, MBBS
  Gopal Bhardwaj, MD
  Gurinderjeet Singh, MD
  Sadbhavna Pandit, MD
  Vidushi Mahajan, MD
  Vishal Guglani, MD
  Goldy Chhabra, MD
Makunda Christian Hospital, Assam
  Roshine Mary Koshy, MD
  Vijayanand Ismavel, MS, MCH
  Shajin T., MD, DNB
  Kenningpeule D. Haikube, MSc
  Jasmine Susan Koshy, MSc
Chinchpada Christian Hospital, Navapur, Maharashtra
  Ashita Singh, MD, MA
  Alice Hepzibah, CH, RN, RM, MSc, FHA
Lady Willingdon Hospital, Manali, Himachal Pradesh
  Anna P. Alexander, MD
  Pradeep Zachariah, MS
  Christina Dhas Sankhro, MBA
  Dennis Martin David, MS
  Philip Alexander, MS
Rural Development Trust Hospital, Anantapur, Andhra Pradesh
  Dasaratha Ramaiah Jinka, MD
  Raghuprakash Reddy Nayakanti, MD
Duncan Hospital, Raxaul, Bihar
  Sheena Evelyn Ebenezer, MD
  Mathew Santosh Thomas, MD
Indian Council of Medical Research-National Institute of Epidemiology, Chennai, Tamil Nadu
  Manoj Muhrekar, PhD
  Elangovan A., PhD

## TIER 3 LABORATORY-BASED SURVEILLANCE

All India Institute of Medical Sciences, New Delhi
  Arti Kapil, MD
  Priyanka Sharma, PhD
  Rakesh Lodha, MD
  Manish Soneja, MD
  Seema Sood, MD
  Bimal Kumar Das, MD
  Kamal Kataria, MS, FACS, FIAGES
St John’s Medical College and Hospital, Bengaluru, Karnataka
  Savitha Nagaraj, MD, DNB
  Vani Channareddy, MD
  Sanju Jose, MSc
  Lissy P. J., Dip Gen Nursing
  Ratnamala Choudhury, MD
  Poornima R. N., MBBS, DNB
  Anirudh V., MS
  Prasanna Kumar A. R., MS, MCh
Postgraduate Institute of Medical Education and Research, Chandigarh
  Pallab Ray, MD, DipNB
  Manisha Biswal, MD
  Archana Angrup, MD
Chacha Nehru Bal Chikitsalya, New Delhi
  Karnika Saigal, MD, DNB
  Deepika Gupta, PhD
  Arnab Ghosh, MD
  Diganta Saikia, MD
  Mamta Senger, MD
Christian Medical College, Vellore, Tamil Nadu
  Shalini Anandan, MD
  Anushree Amladi, MD
  Francis Yesurajan, PhD
  Dhanabhagyam Krishna, MSc, MPhil
  Yamuna D. Bhakthavatchalam, MSc
  Beebi Ezekiel, Dip Gen Nursing
Kanchi Kamakoti Childs Trust Hospital, Chennai, Tamil Nadu
  Sulochana Putli Bai Perumal, MD
  S. Balasubramanian, MD
  S. Senthilnathan, MD
Topiwala National Medical College and BYL Nair Charitable Hospital, Mumbai
  Jayanthi Shastri, MD
  Anuradha De, MD
  Sachee Agrawal, MD
  Sushma Mallik, MD
  Mala Kaneria, MD
Christian Medical College, Ludhiana, Punjab
  Maria Thomas, MD
  Dhruva Ghosh, MS, MCh
  Aroma Oberoi, MD

## COSTING SUBSTUDY

Postgraduate Institute of Medical Education and Research, Chandigarh
  Shankar Prinja, MD, PhD
  Akashdeep Singh Chauhan, PhD
  Saroj Kumar Rana, MSc
  Atul Sharma, PhD

## LABORATORY COORDINATION

Christian Medical College, Vellore
  Balaji Veeraraghavan, MD, PhD
  Agila K. Pragasam, MSc, PhD
  Jobin J. John, PhD
  Shalini Anandan, MD
  Anushree Amladi, MD
  Yamuna D. Bhakthavatchalam, MSc
  Dhiviya P. M. Sethuvel, MSc
  Baby S. Abirami, MSc

## STUDY COORDINATION

Gagandeep Kang, MD, PhD
Jacob John, MD, PhD
Arun S. Karthikeyan, PhD
Karthikeyan Ramanujam, MSc
Venkata Raghava Mohan, MD
Swathi Krishna N., MD
Miriam T. George, PhD
Reshma Raju, MD
Dilesh Kumar A., MPH
Santhosh Kumar G., MPhil
Nikhil Sahai, BTech
Prasanna Samuel, PhD
Nirmal Kumar, MSc
Dilip Abraham, MD, DTM&H
Jayaprakash Muliyil, MD, DrPH

## EXTERNAL COLLABORATORS

Nicholas Grassly, DPhil, Imperial College London, UK
Jason Andrews, MD, Stanford University School of Medicine, USA
Nathan C. Lo, MD, PhD, University of California, San Francisco, USA
Peter Smith, PhD, London School of Hygiene and Tropical Medicine, London, UK
Thomas Cherian, MD, MM Global Health, Geneva, Switzerland

## ACKNOWLEDGMENTS

**Coordination with surveillance networks**
Supriya Kumar, MPH, PhD, Bill and Melinda Gates Foundation, Seattle, USA
Megan Carey, MSPH, Bill and Melinda Gates Foundation, Seattle, USA
Duncan Steele, PhD, Bill and Melinda Gates Foundation, Seattle, USA
Anita Zaidi, MD, MTPH, Bill and Melinda Gates Foundation, Seattle, USA
Rob Breimen, MD, Emory University, USA
Adwoa D. Bentsi-Enchill, MBchB, World Health Organisation, Switzerland
**Editorial support**
Amrita Sekhar, Translational Health Science and Technology Institute, New Delhi

